# Supplementary figures and images for: Impact of pH and protein hydrophobicity on norovirus inactivation by heat-denatured lysozyme
Source: PLoS One. 2020 Aug 19;15(8):e0237888. doi: 10.1371/journal.pone.0237888 (PMC7437907; doi:10.1371/journal.pone.0237888)

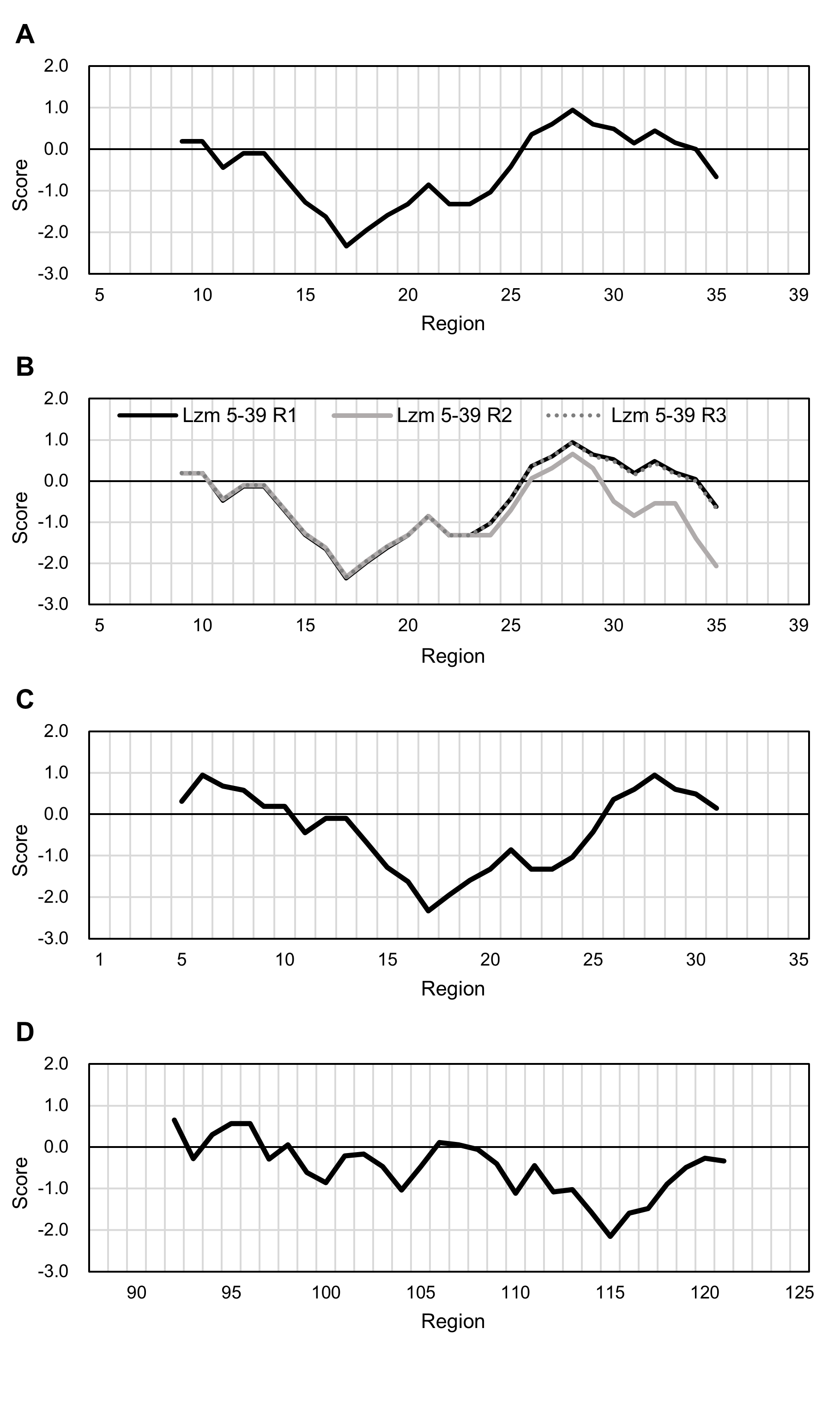

Supplement: S1 Fig — Hydropathicity was computed by ExPASy [16] ProtScale using the Kyte & Doolittle scale [15]. (A) Lzm 5–39, (B) Lzm 5–39 variants (R1, R2, and R3), (C) Lzm 1–35, and (D) Lzm 88–125. (TIF) [file pone.0237888.s001.tif]

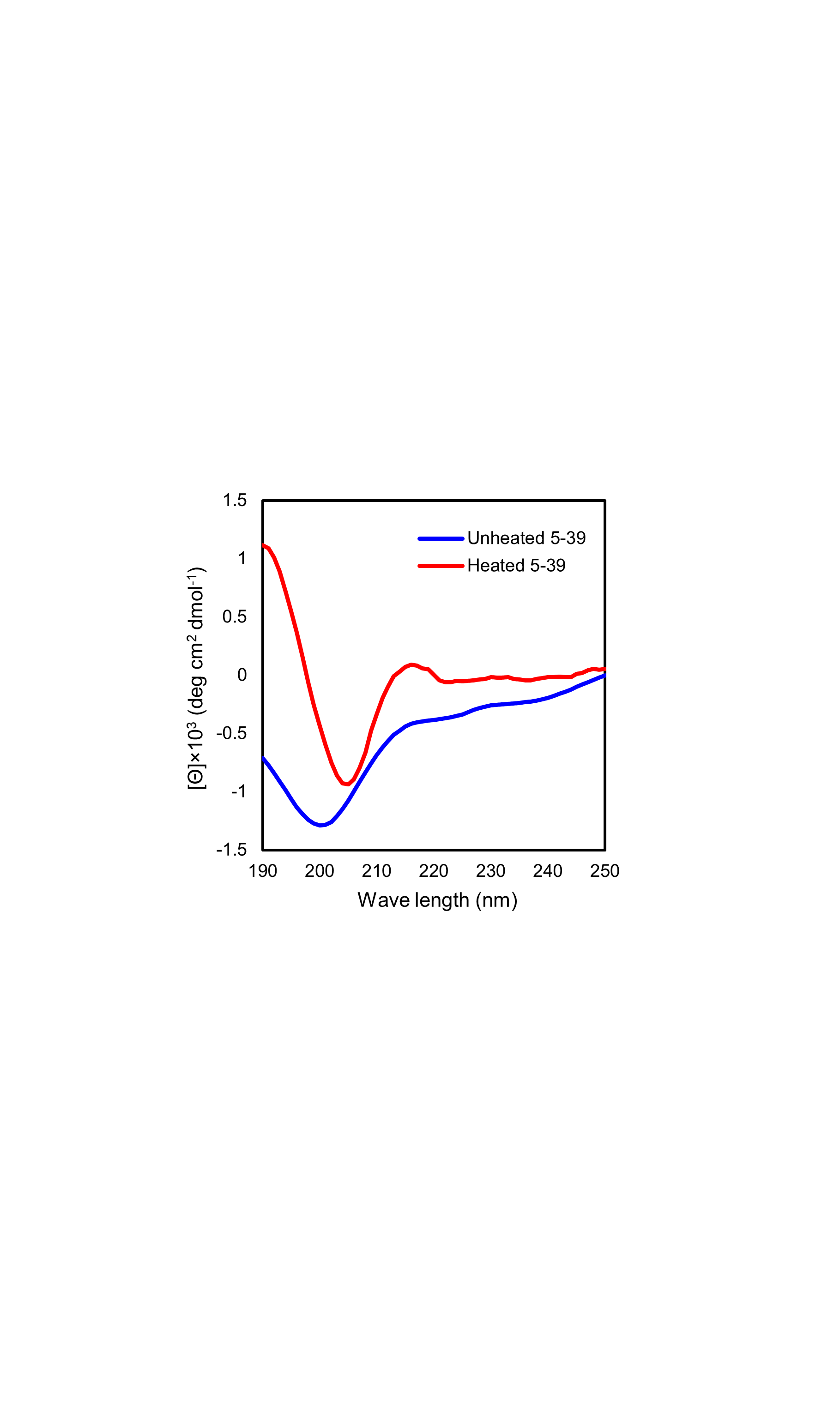

Supplement: S2 Fig — Peptide concentration was adjusted to 900 μM and heat-denatured at 100°C for 40 min (Heated 5–39) or not (Unheated 5–39). The spectra are an average of triplicate measurements. (TIF) [file pone.0237888.s002.tif]
